# Bilateral Visual Impairment following Combination Chemotherapy with Carboplatin in Patients with Small Cell Lung Cancer: A Case Report

**DOI:** 10.3390/medicina60060992

**Published:** 2024-06-17

**Authors:** Jaeha Kim, Junwoo Lee, Seungyeon Lee, Kiyoung Kim

**Affiliations:** Department of Ophthalmology, Kyung Hee University Hospital, Kyung Hee University, Seoul 02447, Republic of Korea

**Keywords:** carboplatin, chemotherapy, cytotoxic drugs, ischemic optic neuropathy

## Abstract

*Background:* Platinum-based combination chemotherapy, including cisplatin and carboplatin, are important cytotoxic anti-cancer agents that are widely used to treat various solid tumors. Carboplatin has a similar effect on survival in small cell lung cancer, but generally has a milder toxicity profile when compared with cisplatin. Both may cause moderate or severe neurotoxicity, but ocular neurotoxicity from carboplatin is rarely reported. *Case presentation:* A 79-year-old man underwent intravenous polychemotherapy (atezolizumab, etoposide, and carboplatin) for small cell lung cancer. One week after the second cycle of chemotherapy, he reported bilateral visual loss as hand motion in both eyes. Dilated fundus examination showed retinal arterial narrowing without hemorrhage, and diffuse choroidal and retinal thinning was observed in an optical coherence tomography scan. Fluorescein angiography revealed significantly delayed circulation without evidence of obstructive lesions. 30-Flicker electroretinogram testing showed a complete absence of cone response in both eyes. The patient’s visual acuity aggravated to no light perception in both eyes, even after the cessation of chemotherapy. *Conclusions:* Carboplatin combination chemotherapy administered at therapeutic doses can result in irreversible visual loss, a side effect that is not widely acknowledged. When using carboplatin, physicians should be aware of its potential ocular toxicity.

## 1. Introduction

Platinum-based agents, which are alkylating cytotoxic agents, form the foundation of chemotherapy for various cancers, including advanced ovarian cancer and advanced metastatic non-small cell lung cancer [1]. The most commonly used drugs in this class are cisplatin and carboplatin. These drugs work by forming DNA cross-links, disrupting DNA function, and inducing apoptosis in tumor cells [2]. The toxicity of platinum drug treatment results directly from the covalent binding of cisplatin and carboplatin to DNA purine bases and indirectly from the drug-induced increase in oxidative stress when scavenging systems are engaged to form stable adducts with the drug [3]. The liver, heart, kidneys, auditory system, and peripheral nerves are the organs most commonly affected by drug toxicity. However, considering the modality of cisplatin-induced cell damage, all organs can potentially be affected to some extent [4]. Numerous previously reported ocular complications have included papilledema, retrobulbar neuritis, optic neuritis, transient cortical blindness, homonymous hemianopsia, and macular pigmentary changes [3,4]. It is known that cisplatin can enter the central nervous system by disrupting the blood–brain barrier due to inflammation or oxidative stress, producing axonal lesions in the optic nerve [4].

Carboplatin/etoposide is an active combination in small cell lung cancer. It has a better toxicity profile than cisplatin/etoposide when compared in non-small cell lung cancer [5]. Generally, carboplatin is said to have milder side effects than cisplatin, whose ocular and orbital toxicity are well known [6].

## 2. Case Presentation

A 79-year-old male with small cell lung cancer stage 4 with multiple intra-abdominal metastasis received two cycles of chemotherapy (atezolizumab, etoposide, and carboplatin) administered over a 10-week period. One week after the second cycle of chemotherapy, he complained of gradually worsening bilateral vision impairment. The dose of carboplatin administered per cycle was approximately 200 mg/m^2^, considering the patient’s overall condition, totaling 710 mg over two cycles. The patient had a history of hypertension but no other significant medical history, except for cataract surgery in the left eye six months ago.

**Figure 1:**
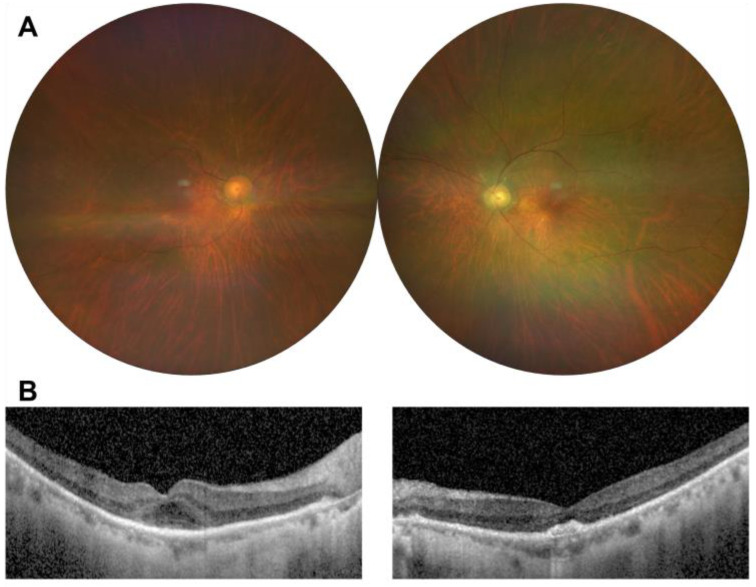
Fundus photographs and optical coherence tomography (OCT) images at the initial outpatient visit. (**A**) Retinal artery narrowing and sclerosis are observed in both eyes, with more severe manifestations in the peripapillary area. (**B**) Optical coherence tomography (OCT) revealed generalized choroidal and retinal thinning in both eyes. Mild cystoid macular edema was present in the right eye, while subfoveal deposition was observed in the left eye.

**Figure 2:**
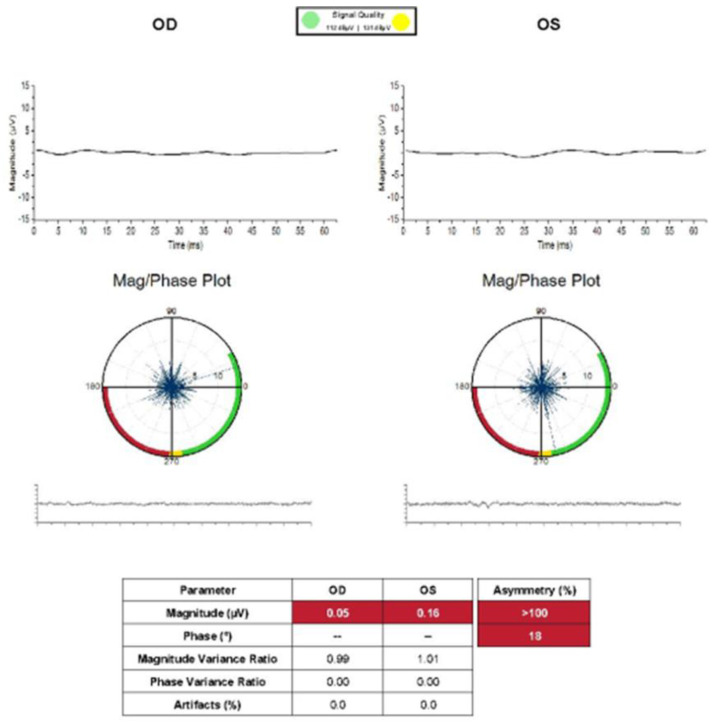
30−Flicker electroretinogram (ERG) findings at the initial outpatient visit. Waveforms were flat in both eyes without a flicker response.

**Figure 3:**
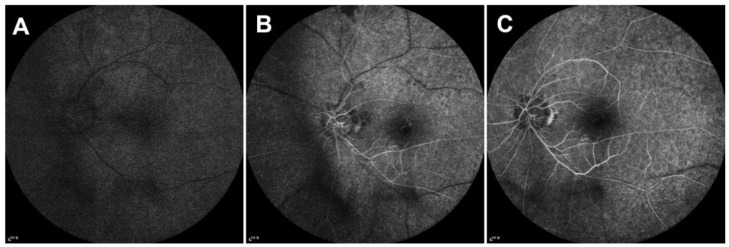
Fluorescein angiography (FA) findings at the initial outpatient visit. (**A**) Choroidal phase: 28 s; (**B**) arterial phase: 34 s; (**C**) venous phase: 44 s. Fluorescein angiography (FA) demonstrated significant delayed filling of the choroidal and arterial circulation and the abnormal delayed drainage of venous circulation in the inferior arcade. Multiple narrowed retinal arteries were observed, although there was no evidence of arterial obstruction.

On ophthalmic examination, the best-corrected visual acuity (BCVA) was hand motion in both eyes, and intraocular pressures were measured at 19 mmHg in the right eye and 18 mmHg in the left eye. There was no relative afferent pupillary defect, and the fundus examination showed optic nerve pallor in the left eye and bilateral retinal arterial narrowing with sclerosis; however, retinal hemorrhage, cotton wool spot, or neovascularization were not observed (Figure 1A). Optical coherence tomography (OCT) revealed generalized choroidal and retinal thinning in both eyes and mild cystoid macular edema in the right eye (Figure 1B). 30-Flicker electroretinogram testing revealed a completely absent cone response in both eyes (Figure 2). Fluorescein angiography demonstrated significant delayed filling of the choroidal and arterial circulation and abnormal delayed drainage of venous circulation in the inferior arcade (Figure 3). There was no evidence of obstructive lesions of arteries or veins. The patient’s blood routine test, coagulation function, and orbital magnetic resonance imaging (MRI) did not reveal any abnormalities. A diagnosis of ischemic optic neuropathy secondary to carboplatin administration was made. To detect potential complications of chemotherapy, close monitoring was decided upon, and further chemotherapy was planned to be temporarily discontinued pending oncological consultation. The patient’s visual acuity aggravated to no light perception even after the cessation of chemotherapy.

## 3. Discussion

It is not uncommon for the initial detection of drug toxicity to take place within the ophthalmology clinic. Advances in multimodal imaging test can facilitate the determination of the likelihood of toxicity associated with each medication, especially in cases where patients receive multiple chemotherapy medications with potential retinal or neurotoxic effects. In this case, the chemotherapy regimen consisted of atezolizumab, etoposide, and carboplatin. Previous reports have not linked atezolizumab and etoposide to retinal toxicity with ischemia. Cisplatin has been associated with several ocular side effects, including causing vaso-occlusive pathologies such as central retinal artery occlusion, cilioretinal artery occlusion, optic neuritis, or optic nerve ischemia [7]. Carboplatin has also been reported to cause ocular side effects, although less frequently [8], but previous reports have shown that carboplatin is associated with severe and irreversible visual loss early in treatment [9]. Although the patient received panretinal photocoagulation in combination with chemotherapy using paclitaxel and carboplatin, there was still a progression of retinopathy leading to severe vison impairment [10]. Fischer et al. [11] reported bilateral papilledema during the treatment of ovarian cancer with carboplatin. Lewis et al. [12] documented unilateral optic disk swelling following carboplatin chemotherapy for ovarian cancer. Recently, Shihadeh et al. [13] described a case of carboplatin-induced bilateral optic neuropathy with irreversible vision loss in a patient with bladder cancer. Maleki et al. [14] also reported carboplatin-induced bilateral optic neuropathy in a patient with metastatic squamous cell carcinoma of the tongue, resulting in no light perception in the left eye.

The cause of retinal ischemia induced by cisplatin/carboplatin is uncertain, but various mechanisms have been proposed. Excessive oxidative stress is believed to be the cause of retinal toxicity [15]. In vitro studies have indicated a link between cisplatin-induced platelet activation and the activation of phospholipase A2, which may explain the thrombotic side effects of cisplatin [16]. Some studies have identified microvascular thrombosis as the cause of visual loss in patients receiving chemotherapy with cisplatin and carmustine [17]. Another hypothesis suggests that decreased renal excretion may lead to drug accumulation in the central nervous system [18]. In ERG assessments, individuals treated with intravenous cisplatin for germinal cell cancer exhibited notably diminished b-wave amplitudes and prolonged a-wave implicit times during isolated cone response testing with 30 Hz flickering white light [19]. Regarding VEP, cisplatin-induced retinopathy correlates with heightened implicit times and diminished amplitude [20]. Notably, the impact on VEP appears to be unrelated to the cumulative dose of cisplatin administered [21].

This case report has several limitations. Firstly, due to the absence of ophthalmic records prior to anticancer treatment, we were unable to directly prove that the vision loss occurred abruptly following the anticancer therapy. Therefore, we could not completely rule out the possibility of underlying conditions such as bilateral diffuse uveal melanocytic proliferation, cancer-associated retinopathy, and non-paraneoplastic autoimmune retinopathy. Secondly, while previous reports have not linked atezolizumab and etoposide with retinal toxicity and ischemia, this does not conclusively rule out their potential contribution to visual loss, especially given their concurrent administration with carboplatin.

## 4. Conclusions

Ocular complications reported after the use of carboplatin have typically been associated with severe retinal ischemic changes and vision impairment. However, this case can be considered the first report showing irreversible visual loss accompanied by optic nerve pallor and sclerotic retinal artery without any other evident ischemic signs on ophthalmic examination. This case highlights the need for oncologists and ophthalmologists to be aware of the potential ocular side effects of carboplatin.

## Data Availability

The data generated in the present study are included in the figures of this article. The data can be obtained from the authors upon making a reasonable request.
